# THE POTENTIATION OF THE RADIOPROTECTIVE EFFICACY OF TWO MEDICAL COUNTERMEASURES, GAMMA-TOCOTRIENOL AND AMIFOSTINE, BY A COMBINATION PROPHYLACTIC MODALITY

**DOI:** 10.1093/rpd/ncw223

**Published:** 2016-12-23

**Authors:** Vijay K. Singh, Oluseyi O. Fatanmi, Stephen Y. Wise, Victoria L. Newman, Patricia L.P. Romaine, Thomas M. Seed

**Affiliations:** 1 Department of Pharmacology and Molecular Therapeutics, F. Edward Hébert School of Medicine ‘America's Medical School’, Services University of the Health Sciences,Bethesda, MD, USA; 2 Armed Forces Radiobiology Research Institute, Uniformed Services University of the Health Sciences , Bethesda, MD, USA; 3 Tech Micro Services, Bethesda, MD, USA

## Abstract

This study was designed to evaluate the possible potentiation of survival protection afforded by relatively low-dose amifostine prophylaxis against total body irradiation in combination with a protective, less toxic agent, gamma-tocotrienol (GT3). Mice were administered amifostine and/or GT3, then exposed to 9.2 Gy ^60^Co γ-irradiation and monitored for survival for 30 days. To investigate cytokine stimulation, mice were administered amifostine or GT3; serum samples were collected and analyzed for cytokines. Survival studies show single treatments of GT3 or amifostine significantly improved survival, compared to the vehicle, and combination treatments resulted in significantly higher survival compared to single treatments. *In vivo* studies with GT3 confirmed prior work indicating GT3 induces granulocyte colony-stimulating factor (G-CSF). This approach, the prophylactic combination of amifostine and GT3, which act through different mechanisms, shows promise and should be investigated further as a potential countermeasure for acute radiation syndrome.

## INTRODUCTION

The threat of a nuclear disaster is a critical concern and a top priority for all US agencies involved in domestic security and public health preparedness. In 2006, Congress passed the Pandemic and All-Hazards Preparedness Act to broaden and modify our national preparedness effort^(1)^. As made evident by the BioShield legislation, the need for new countermeasures that are safe, easily administered and effective at reducing or eliminating the public health impact of acute high-dose radiation are urgently needed. In the event of radiological or nuclear materials exposure, medical care would be focused on treating exposure casualties with acute radiation syndrome (ARS)^(2, 3)^. Inherent to this ‘national preparedness effort’ is a complementary effort to protect via appropriate countermeasures both first responders and military personnel tasked to enter ‘hot’ nuclear/radiological areas in order to locate, medically assist and evacuate casualties and to maintain civil order.

Vitamin E, a fat-soluble nutrient, is especially well known for its antioxidant, neuroprotective and anti-inflammatory properties^(4, 5)^. Vitamin E represents a family of compounds, which act as important antioxidants that regulate peroxidation reactions and control free-radical production within the body. This family of compounds has eight different isoforms that belong to two categories: four saturated analogs (α, β, γ, and δ) called tocopherols and four unsaturated analogs referred to as tocotrienols^(5)^. These eight components are collectively known as tocols. Gamma-tocotrienol (GT3) is a potent inhibitor of 3-hydroxy-3-methylglutaryl-coenzyme A (HMG-CoA) reductase. Its antioxidant activity was a compelling reason to evaluate it for radioprotective efficacy; in recent years, it has received a great deal of attention by researchers and appears to be one of the most promising radioprotective tocols tested to date. Recent results suggest that GT3-stimulated granulocyte colony-stimulating factor (G-CSF) is involved in its radioprotective mechanism^(6, 7)^.

Amifostine (WR2721, Ethyol), 2-(3-aminopropyl) aminoethylphosphorothioate, is a radioprotective agent that has been fully approved for human use by the United States Food and Drug Administration (FDA)^(8–11)^. Despite the FDA's approval, the drug has only been authorized for use for a very narrowly defined medical indication, namely, the reduction of xerostomia (dry mouth) that results from injury of salivary glands in patients undergoing radiotherapy for the treatment of head and neck cancers^(12)^. Because of the performance decrementing side-effects, amifostine has not been approved for general use in radiation protection of high risk personnel. However, it has been demonstrated both experimentally and clinically, that by using sufficiently low doses of amifostine prophylactically, its toxic side-effects can be minimized while still maintaining significant levels of radioprotection.^(11)^ Therefore, based on the results published previously using lower doses of GT3 and amifostine, it appeared logical to test whether a low dose of amifostine could enhance the radioprotective efficacy of low dose of GT3 by acting through, separate, but complementary protective pathways. The development and promulgation of such prophylactically applied radiation countermeasures would be exceedingly useful for military personnel and first responders to nuclear/radiological contingencies.

Our objective was to investigate whether small doses of two radiation countermeasures can be combined for additive/synergistic efficacy. We selected GT3 and amifostine based on their radioprotective efficacy and different mode of actions. Our results indicate that amifostine at low doses enhanced the efficacy of low doses of GT3 in mice. This study suggests that both agents can be used in combination at lower doses and still achieve optimal protection against a lethal dose of radiation without producing adverse effects (toxicity).

## MATERIALS AND METHODS

### Mice

Male 6–8 week-old CD2F1 mice were purchased (Harlan Laboratories, Inc., Indianapolis, IN, USA) and housed (8 per cage) in an environmentally controlled facility accredited by the Association for Assessment and Accreditation of Laboratory Animal Care-International. All mice were kept in rooms with a 12 h light and 12 h dark cycle. The mouse holding room was maintained at 20–26°C with 10–15 air exchange cycle/h and a relative humidity of 30–70%. Upon arrival, the mice were held in quarantine for 1 week. A microbiological examination of representative samples ensured the absence of *Pseudomonas aeruginosa*. Mice were provided certified rodent rations (Teklad Rodent Diet, Harlan Laboratories, Inc.) and acidified water (pH = 2.5–2.8) *ad libitum*. All animal procedures were performed according to a protocol approved by the Armed Forces Radiobiology Research Institute's (AFRRI) Institutional Animal Care and Use Committee. Research was conducted according to the Guide for the Care and Use of Laboratory Animals prepared by the Institute of Laboratory Animal Resources, National Research Council, US National Academy of Sciences^(13)^.

### Drug preparation and administration for mice

GT3 formulation in 5% Tween-80 in saline was purchased from Yasoo Health, Inc. (Johnson City, TN, USA). Olive oil was used as vehicle control (equivalent to the quantity of GT3) in 5% Tween-80. The final GT3 concentration was adjusted to administer a dose of 25 or 50 mg/kg in 0.1 ml. Control mice received 0.1 ml of vehicle. Mice were administered GT3 or vehicle subcutaneously (sc) at the nape of the neck with a 23 G needle attached to a Luer-Lock syringe 24 h before irradiation. No infections or local reactions were noted at the site of injection.

Pharmaceutical grade amifostine was purchased from MedImmune (Gaithersburg, MD, USA) as 500 mg sterile lyophilized powder vial and reconstituted with normal saline (0.9% sodium chloride). A dose of 30 or 50 mg/kg was injected (sc) in 0.1 ml, approximately ~30 min prior to irradiation in survival studies. Control groups received equivalent volumes of normal saline.

### Irradiation

Mice were placed in ventilated Plexiglas boxes compartmentalized to accommodate eight mice per box and exposed to bilateral irradiation in the AFRRI ^60^Co facility at a dose rate of 0.6 Gy/min. Animals were irradiated with midline doses of 9.2 Gy (with an estimated LD_50/30_ dose of 8.6 Gy for CD2F1 mice). After irradiation, mice were returned to their cages and monitored for 30 days. Radiation dosemetry was based primarily on the alanine/EPR (electron paramagnetic resonance) system^(14)^, currently accepted as one of the most accurate methods for relatively high radiation doses and one used for intercomparison between national metrology institutions. The calibration curves (spectrometer e-Scan, Burker Biospin, Inc., Madison, WI, USA) used in dose measurements at AFRRI are based on standard alanine calibration sets purchased from the US National Institute of Standards and Technology (NIST, Gaithersburg, MD, USA). The alanine dosemeters obtained from NIST had been calibrated in terms of absorbed dose to water using the US national standard radiation sources. At AFRRI, identical alanine dosemeters were placed midline within mouse phantoms (Plexiglas 1” diameter, 3” length) and irradiated for predefined periods of time. Measurement of their EPR signals using the calibration curve constructed with alanine dosemeters from NIST-provided dose rates to water in the core bodies of mice. A small correction was subsequently applied for the difference in mass energy absorption coefficients between water and soft tissue.

### Blood collection and multiplex luminex analysis of cytokines

Blood was collected from anesthetized (1–5% isoflurane, Abbott Laboratories, Chicago, IL, USA) mice via the inferior vena cava using a 23 G needle. After collection, blood was transferred to Capiject serum separator tubes (Terumo Medical Corp., Elkton, MD, USA), allowed to clot for 30 min at room temperature, and centrifuged at 400 *g* for 10 min. The serum was collected and stored at –70°C until used. Luminex 200 (Luminex Corp., Austin, TX, USA) was used to detect cytokines, chemokines, and growth factors in mouse serum samples supernatants as described earlier using multiplex kits (Bio-Rad Inc., Hercules, CA, USA)^(15)^. Mice administered amifostine were treated with 50, 100 or 150 mg/kg dose. Serum samples were collected 4, 8, 24 and 48 h post amifostine administration. Cytokines were evaluated in mice treated with 25 and 50 mg/kg of GT3 and blood samples were collected 24 h post-GT3 injection.

### Statistical analysis

For survival data, a log-rank test was used to compare survival curves. Fisher's exact test was used to compare survival rates at the end of 30 days, with a Bonferroni correction used to control for type-I error if multiple comparisons were used. For cytokine data analyses, mean values with standard errors (SE, when applicable) were reported. Analysis of variance (ANOVA) was used to detect whether there were significant differences between experimental groups. When significance was indicated, a Tukey's post-hoc test was used to determine significant differences between particular groups. Equal variance were assumed between treatments. All statistical tests were two-sided with a 5% significance level and performed using the statistical software SPSS version 19 (IBM, Armonk, NY, USA).

## RESULTS

### Radioprotective efficacy of GT3 (50 mg/kg) and amifostine (50 or 30 mg/kg) in irradiated mice

**Figure 1.**
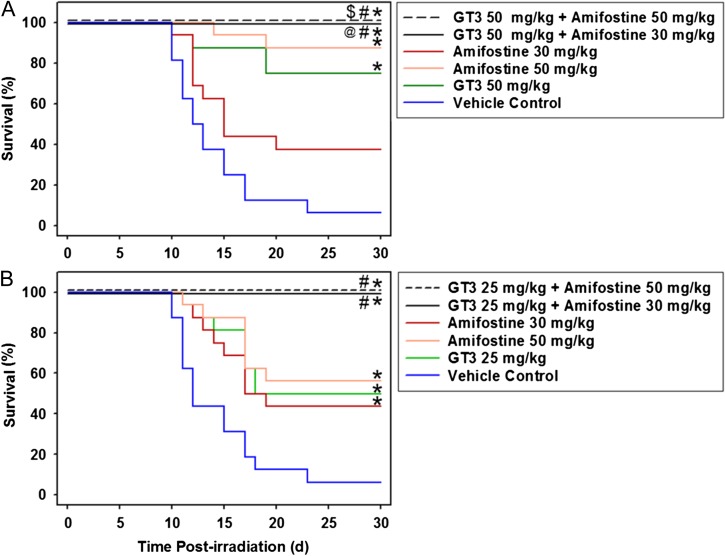
Efficacy of single GT3 (25 or 50 mg/kg) or amifostine (30 or 50 mg/kg) administrations or combination treatment on mouse survival after exposure to 9.2 Gy ^60^Co γ-radiation (0.6 Gy/min). Mice (*n* = 16 per treatment group) were administered GT3 24 h prior to and/or amifostine 15 min prior to radiation exposure. Mice were then observed for survival for 30 days post-irradiation. (**A**) All mice received GT3 50 mg/kg and/or amifostine (30 or 50 mg/kg) prior to irradiation. * Indicates a significant difference compared to vehicle control treated mice; # indicates there is a significant difference compared to amifostine (30 mg/kg) treated mice (*p* < 0.05). (**B**) All mice received GT3 (25 mg/kg) and/or amifostine (30 or 50 mg/kg) * indicates a significant difference compared to vehicle control treated mice; # indicates there is a significant difference compared to GT3 (25 mg/kg) treated mice; $ indicates there is a significant difference compared to amifostine (50 mg/kg) treated mice; @ indicates there is a significant difference compared to amifostine (30 mg/kg) treated mice (*p* < 0.05). For both panels, the two combination treatment groups overlap at 100% survival; all statistical symbols above the line correspond to GT3 50 mg/kg + Amifostine 50 mg/kg, and all those below correspond with GT3 50 mg/kg + 30 mg/kg.

Mice were administered either GT3 or amifostine, or the two in combination, and exposed to 9.2 Gy ^60^Co γ-radiation and subsequently monitored for 30 days. Figure 1A shows that 50 mg/kg GT3 was capable of protecting 75% of mice. The combination of GT3 and amifostine (either 30 or 50 mg/kg) improved survival to 100% of the mice for the full 30 days of the experiment (*n* = 16). Amifostine alone (50 mg/kg) provided protection to 87.5% of mice (Figure 1A) whereas the 30 mg/kg dose provided 37.5% protection (Figure 1A). Both combined treatment groups, GT3-treated groups, and amifostine (50 mg/kg)-treated groups showed significantly increased rates of survival compared to vehicle-treated control (6%). Both combined treatment groups were significantly improved compared to the amifostine (30 mg/kg) treated group.

### Radioprotective efficacy of GT3 (25 mg/kg) and amifostine (50 or 30 mg/kg) in irradiated mice

In initial the experiment (as described above), the GT3 treatment dose of 50 mg/kg was sufficiently high as to minimize a difference between GT3 and combined treatment groups. Therefore, the experiment was repeated, but using a lower treatment dose of 25 mg/kg GT3 in combination with either 30 or 50 mg/kg doses of amifostine. Figure 1B demonstrates the results of the repeat experiment. Both groups (*n* = 16) of mice that received GT3 (25 mg/kg) and amifostine (either 30 or 50 mg/kg), provided 100% survival, which was a significant increase in survival compared to either GT3 alone (50%), to amifostine alone (at both doses of 30 or 50 mg/kg) (44% and 56% survival rates, respectively), or to the vehicle alone-treated group (6%). In sum, all treatments yielded significantly higher survival rates compared to the vehicle control.

### GT3 and amifostine induction of cytokines in mouse model

**Figure 2.**
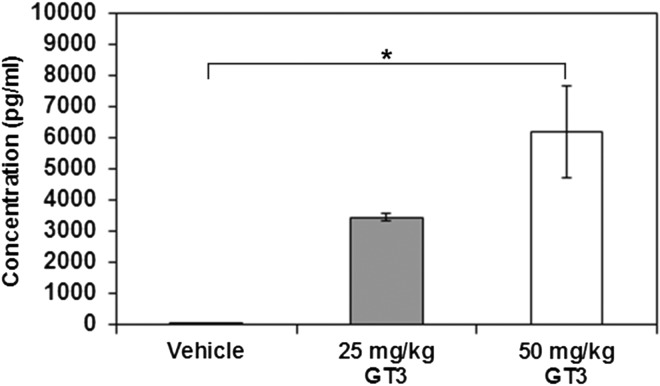
G-CSF levels in mice serum 24 h after GT3 treatment (25 or 50 mg/kg). Mice were administered GT3 or vehicle (sc) and serum samples were collected at 24 h and analyzed using Luminex (*n* = 6). * Indicates that the two groups are significantly different (*p* < 0.05).

To confirm earlier findings of G-CSF induction by GT3, mice were administered GT3 (25 mg/kg or 50 mg/kg) or vehicle. Serum samples were collected 24 h after GT3 injection (*n* = 6) and analyzed using Luminex. GT3-induced G-CSF in both treatment groups; 50 mg/kg of GT3-induced G-CSF levels significantly higher than the vehicle-treated group (Figure 2). Earlier, we have reported induction of various cytokines by GT3 in irradiated and unirradiated mice^(16)^ and others have also reported induction of similar cytokines by GT3 at various time points post-GT3 injection^(17)^. In the above studies, GT3 was used at higher doses, in the current study we demonstrate that lower dose of GT3 which have been combined with amifostine to demonstrate radioprotective efficacy induce significant levels of G-CSF.

**Figure 3.**
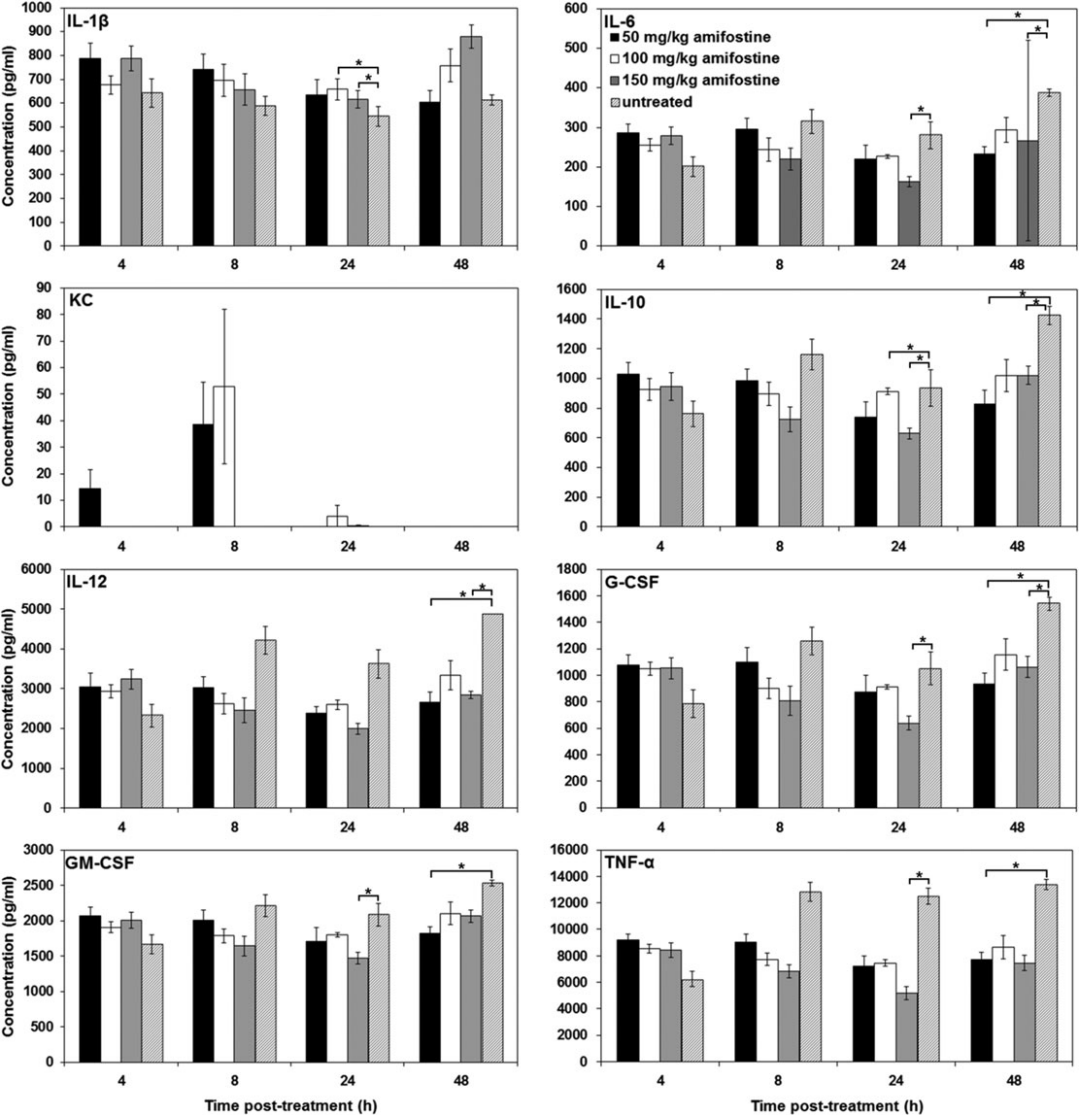
Cytokines induced in the mouse serum at various time points after amifostine administration (sc). Serum samples were collected at various time points (4, 8, 24, and 48 h) after amifostine injection. Samples were stored at −80°C until analysis with Luminex (*n* = 6). * Indicates that the treatment group is significantly different (*p* < 0.05).

To determine whether amifostine induces G-CSF, mice were administered 50, 100, 150 mg/kg amifostine; serum samples were collected at various time points (4, 8, 24 and 48 h) post-treatment (*n* = 6) and analyzed using multiplex Luminex. Initially, we investigated 50 mg/kg dose of amifostine and did not find high levels of G-CSF in serum samples, therefore, did not justify investigation of cytokine induction with lower doses of amifostine. To rule out induction of G-CSF with higher doses of amifostine, we included 100 and 150 mg/kg doses of amifostine. Figure 3 displays the time-dependent changes in serum levels of 8 of the 40 cytokines analyzed following the various treatments; these eight cytokines include interleukin-1β (IL-1β), IL-6, keratinocyte-chemoattractant (KC), IL-10, IL-12, G-CSF, GM-CSF and tumor necrosis factor-α (TNF-α). Amifostine administration significantly inhibited certain cytokines at 24 h (IL-6, IL-10, G-CSF, GM-CSF and TNF-α) or 48 h (IL-6, IL-10, IL-12, G-CSF, GM-CSF and TNF-α). Only IL-1β was significantly stimulated at 24 h after amifostine administration. We tested 50 mg/kg dose of amifostine in combination with 50 mg/kg of GT3 at 24 h and combination treatment did not show any enhancement of GT3-induced G-CSF (data not presented).

## DISCUSSION

In rodent models of acute radiation syndrome (ARS), radiation dose reduction factors (DRFs) for amifostine delivered over a full range of tolerated doses have been estimated to range from 1.6 to 3.0 for the hematopoietic syndrome and 1.6–2.1 for GI syndrome^(8)^. These relatively high DRFs have not been observed with any other radiation countermeasure under development^(18, 19)^. Under standard clinical dosing regimens, amifostine is generally well tolerated by the majority of patients; serious side-effects are rare, however, minor toxic responses occur frequently that mainly include but not limited to nausea, vomiting and adverse cutaneous reactions following sc injection. It is because of these toxic side-effects, amifostine has not been approved for general use as a radioprotector^(18)^. Nevertheless, alternative indications have been proposed for amifostine including: (a) global cytoprotection with significant survival benefit when administered at high drug doses, notwithstanding the potential risks of toxic side-effects, (b) selective protection of specific progenitorial tissue compartments at low drug doses^(11)^, and (c) protection against late-arising, radiation-induced cancers with low drug doses^(20)^. Despite the modest advancements in maximizing amifostine's radioprotective utility, none of the strategies to date have succeeded in making amifostine more amenable for general use; i.e. by minimizing amifostine's toxic side-effects while retaining its full radioprotective potential.

The radioprotective potential of antioxidants and free radical scavengers, such as amifostine, is derived from their ability to reduce levels of reactive oxygen species which are induced by irradiation, thus decreasing DNA damage, lipid peroxidation, and damage by chemical modification^(21)^. Recently, we determined that the effective radioprotective, non-toxic minimum dose of amifostine, when administered sc to mice, appears to lie between 25 and 50 mg/kg^(11)^. This finding prompted us to evaluate low dose of amifostine in combination with a low dose of another radiation countermeasure acting through a different mode of action.

Recently, we also reported that the radioprotective efficacy of GT3 is mediated through G-CSF stimulation and use of G-CSF antibody completely abrogates the radioprotection of GT3^(16)^. G-CSF antibody administration also exacerbated the radiation-induced injury in mice^(22)^ suggesting the radioprotective role of G-CSF. G-CSF is an agent which has been evaluated for efficacy against radiation injuries in animal models across four species^(18, 23–26)^. G-CSF, as well as PEGylated G-CSF, have recently been approved for hematopoietic ARS treatment by the FDA^(27–29)^. Under such situation, it appeared logical to test whether low dose of GT3 could enhance the radioprotective efficacy of a minimally toxic, low dose of amifostine. Hence, we combined GT3 (25 or 50 mg/kg) with two different low doses of amifostine (30 or 50 mg/kg) for protecting mice exposed to whole-body ^60^Co γ-irradiation. Our results demonstrate that radioprotective efficacy of both agents can be enhanced significantly by combining low doses of both agents (Figure 1). Consistent with all our previous radiation countermeasure studies spanning ten plus years, we have used a dose rate of 0.6 Gy/min; however, higher dose rates may occur during a radiological or nuclear event which may result in different biological sequela.

A dose of 500 mg (absolute) of amifostine administered sc to human patients before each fraction of radiotherapy is well tolerated by the majority of patients (85%). The remaining 15% had occurrences of cumulative asthenia and or a fever/rash reaction. At this dose, mild nausea was frequent (29%), but instances of hypotension were minor (3%)^(30, 31)^. The dose administered to mice in this study and its relative human equivalent dose is much lower than the dose administered to humans undergoing radiotherapy. There are reports demonstrating that lower doses of amifostine elicit lower rates of adverse effects in respect to emesis, hypotension, somnolence and sneezing in animal models as well as in human^(12, 32–34)^.

There is a recent report of combining amifostine with metformin (1,1-dimethylbiguanide hydrochloride) to assess protective effects of the combination on spleen colonies in irradiated C3H mice^(35)^. In this study a higher dose of amifostine (400 mg/kg) was used, and there was no improvement in the group receiving combined treatment compared with metformin alone. In this study, both drugs were administered 24 h after irradiation since the objective of this study was to identify countermeasures which can be administered as a radiomitigator. In our study, we have administered significantly lower doses of amifostine (30 or 50 mg/kg) 30 min prior to irradiation to display radioprotective properties.

In large part, the focus on cytokines and growth factors has been based on their potential to act as radiomitigators to enhance recovery of the hematopoietic system from radiation damage, as demonstrated in multiple *in vitro* and *in vivo* models^(36, 37)^. As stated above, two growth factors have been approved for treatment of hematopoietic ARS^(27–29)^, some cytokines have received FDA approval for treatment of neutropenia and thrombocytopenia caused by anti-cancer radiotherapy and chemotherapy, and several are in development^(18, 24, 38, 39)^. However, when used as single agents, these specific factors have limited efficacy in the medical management of ARS. Nevertheless, we demonstrate here in these studies a successful new strategy to improve basic radioprotective treatments using amifostine; namely, the simple combination of low doses of two distinct types of antioxidant/free radical scavenging protectants, one of which mitigates injury through an induced G-CSF pathway, into a single treatment modality.

Earlier we have demonstrated that a number of radiation countermeasures currently under investigation stimulate G-CSF production in mice, including CBLB502 in nonhuman primates in addition to mice and canines^(15, 16, 40–43)^. Further, the radioprotective efficacy of several of these agents was abrogated by administration of G-CSF antibody^(15, 16, 41–44)^, suggesting that radioprotective efficacy of these agents is mediated through G-CSF. G-CSF and IL-6 have also been identified as efficacy biomarkers for CBLB502^(43)^. Current results of G-CSF induction by GT3 confirms earlier findings^(44)^. GT3 stimulates production of G-CSF, IL-6, and KC in mice. Though the role of G-CSF in regards to radioprotection has been explored and confirmed, IL-6’s role remains an enigma and needs further investigation^(24, 45, 46)^.

Our observations suggest that different pathways are involved in radioprotection by amifostine and GT3. Amifostine failed to stimulate cytokine production in the mouse model or in an *in vitro* system using cells of human origin (data not presented). Although GT3-induced G-CSF may not be directly responsible for GT3’s radioprotective abilities, it is indeed associated temporally and pathophysiologically. However, it is more than likely that G-CSF triggers pathways that differ from those involved in amifostine radioprotection. GT3 is an HMG-CoA (3-hydroxy-3-methylglutaryl-coenzyme A) reductase inhibitor; it was demonstrated that GT3 decreased radiation-induced vascular oxidative stress, an effect that was reversible by mevalonate (the product of the reaction catalyzed by HMG-CoA)^(47)^. GT3 reduced intestinal radiation injury and accelerated the recovery of soluble markers of endothelial function^(47, 48)^. GT3 also ameliorated endothelial cell apoptosis, reduced endothelial cell guanosine triphosphate cyclohydrolase 1 (GTPCH) feedback regulatory protein (GFRP) levels and GFRP-GTPCH binding by decreasing transcription of the GFRP gene. The thrombomodulin-activated protein C pathway plays an important role in mitigation of whole-body irradiation-induced mortality^(49)^. This may be critical for the HMG-CoA reductase inhibition-dependent radiation protection by GT3.

Amifostine, the FDA-approved and systemically active radioprotectant, works through a variety of mechanisms. The most immediate protection is provided by free radical scavenging and the neutralization of reactive metabolites through the donation of hydrogen; hydrogen donation by the thiol group may also aid in accelerating DNA repair^(50)^. Additional studies have shown that amifostine inhibits apoptosis, alters cell cycle progression, and provides cytoprotection through the p53 protein pathway^(51)^. Amifostine also up-regulates hypoxia inducible factor-1α (HIF-1α) which promotes intracellular hypoxic conditions, ultimately reducing reactive oxygen species levels at the time of radiation exposure. HIF-1α also aids in regulating glycolysis, angiogenesis and apoptosis and has been linked with radioresistance^(52)^. Additional reports include altered gene expression and modified enzyme activity as mechanisms by which amifostine provides radioprotection. Studies support the concept that protection of acute radiation injury by amifostine can only be achieved if it is present during the initial radiological events.

In summary, our preliminary results demonstrate that low doses of GT3 and amifostine can be combined to achieve optimal radioprotection while reducing and potentially eliminating unwanted side-effects. It will be appropriate to test the efficacy of the amifostine and GT3 combination, using the mouse model over an expanded range of circumstances (i.e.  hematopoietic and gastrointestinal syndromes, combined injury and mixed field (neutron/gamma exposure)). It is important to note that both countermeasures used in this study are radioprotectors (requiring administration prior to radiation exposure) and relevant to military or civilian population with a known and imminent radiation threat. These agents do not provide significant protection when used as radiomitigators (administered after radiation exposure). Currently, we are planning to determine DRF, complete blood counts, and colony forming unit-spleen survival after irradiation in mice receiving combined treatment. We expect this combination will be moved into advanced development in the near future and may prove to be optimal for ARS in humans.
